# A ‘Vocal Locals’ social network campaign is associated with increased frequency of conversations about mental health and improved engagement in wellbeing-promoting activities in an Australian farming community

**DOI:** 10.1186/s12889-024-18193-7

**Published:** 2024-03-02

**Authors:** Chloe M. E. Fletcher, Dale Woolford, John Gladigau, Kate M. Gunn

**Affiliations:** 1 IIMPACT in Health, Department of Rural Health, Allied Health and Human Performance, University of South Australia, Adelaide, SA 5000 Australia; 2 Gladigau Enterprises Pty Ltd, Loxton, SA 5333 Australia

**Keywords:** Community, Coping, Farming, Mental health, Mental health intervention, Rural, Social networks, Stress, Wellbeing

## Abstract

**Background:**

Farmers face numerous barriers to accessing professional mental health services and instead report a preference for informal support systems, such as lay or peer networks. Farmers also experience barriers to investing time in maintaining or improving their wellbeing, stemming from sociocultural norms and attitudes that are widespread in agricultural communities. The Vocal Locals social network campaign is an ifarmwell initiative that aims to promote conversations about wellbeing and challenge attitudes and behaviours that contribute to farmers’ poor mental health.

**Methods:**

The Vocal Locals campaign was underpinned by the socio-ecological model which explains human behaviour as stemming from interactions between the individual, their closest social circle, the community, and broader society. The campaign ran in Loxton, South Australia, from June to August 2022. Ten community members (8/10 farmers) became ‘Vocal Locals’ and were supported to share ‘calls-to-action’ to encourage people in their social networks to engage in wellbeing-promoting activities. A broader communications campaign reinforced key messages and amplified Vocal Locals’ activities in the community. The intrapersonal and community-level impacts of the campaign were evaluated via pre- and post-campaign surveys of Vocal Locals and community members respectively.

**Results:**

Vocal Locals reported significantly lower psychological distress (*p* = .014), and higher positive mental wellbeing (*p* = .011), levels of general mental health knowledge (*p* = .022), and confidence helping someone with poor mental health (*p* = .004) following the intervention. However, changes in stigmatising beliefs about mental illness, confidence recognising poor mental health, and confidence and comfort speaking to others about mental health were non-significant. Community members who were familiar with the campaign reported having significantly more wellbeing-related conversations post-campaign compared to before (*p* = .015). Respondents also reported being more comfortable speaking to others about mental health or wellbeing (*p* = .001) and engaging more in activities to maintain or improve their wellbeing (*p* = .012) following the campaign.

**Conclusions:**

The Vocal Locals social network campaign is an example of how science and community can be brought together to achieve meaningful outcomes. The campaign may serve as a model for others who wish to challenge attitudinal or knowledge-related barriers to help-seeking and improve engagement in wellbeing-promoting activities in difficult-to-reach communities.

**Supplementary Information:**

The online version contains supplementary material available at 10.1186/s12889-024-18193-7.

## Background

The Australian Bureau of Statistics defines remoteness based on the road distances people must travel to access services [1]. According to the Accessibility and Remoteness Index of Australia, around 7 million Australians live in rural and remote areas [2]. These Australians face a range of health inequities compared to those living in major cities [3, 4]. Despite similar rates of self-reported depression and anxiety [5–7], Australians living in outer regional, remote, and very remote areas are more likely to die by suicide [8]. As remoteness increases, so does the rate of suicide; men living in very remote areas are twice as likely to die by suicide than their metropolitan counterparts [9]. A similar pattern has been observed among women [9], albeit at a lower rate than men. The reasons for this are complex. Rural Australians experience geographical and social isolation [10, 11], have reduced access to healthcare services [12], and often work in interdependent industries that are reliant on external factors such as the weather [13]. It has been proposed that lower levels of diagnosis and treatment of mental illness contribute to increased suicide rates in rural areas [14]. This may in part be due to a shortage of mental health care providers in rural areas, where it is often general practitioners who are tasked with diagnosing and treating mental health conditions [15]. Where services are available, reduced utilisation of mental health support services, particularly among young men, is likely to contribute to underdiagnosis [6, 14]. Another factor is likely to be reduced awareness or recognition of mental illness among rural Australians, in themselves and others. For example, Handley et al. [16] found that one-third of rural people with moderate/high distress who were surveyed did not report that they had been experiencing mental health problems.

Suicide rates are higher still among farmers and farm workers, who die by suicide at a rate that is almost 60% higher than non-farming rural people [17]. Poor mental health and suicide among farmers is thought to be associated with complex and interconnected factors including uncertainty and lack of control in farming, relationship problems and breakup, financial difficulties, pending retirement, and access to means [18, 19]. Research comparing farming- and non-farming-related suicides found that farmers were less likely than other rural people to have a diagnosed mental illness or have received mental health support prior to their death [3]. Some researchers have suggested that there may be high rates of unrecognised and untreated depression among farming men [18]. A range of personal and contextual factors are also thought to contribute to a reluctance towards accessing mental health support among farmers. These include sociocultural norms of masculinity, stoicism, and self-reliance [18, 20–24], and negative attitudes towards mental illness, mental health professionals, and seeking help for psychological problems [20, 23, 25–27]. A lack of cultural understanding among rural health professionals [28], combined with social isolation from the broader community [23], may contribute to farmers’ distrust of health professionals and hesitance towards engaging with mainstream mental health services. Long working hours, which appear to increase during times of hardship [13, 24], and prioritisation of farm work and productivity over wellbeing [26], form additional barriers to accessing support.

Further compounding this issue, farming communities are expected to be impacted by increasing frequency and intensity of weather events such as drought, flooding, and bushfires in coming years [29–31]. Prolonged drought, flooding, and bushfires are thought to have serious, negative implications for wellbeing [13, 32–38]. Because farmers rely on the weather for their work and livelihood, it can be reasonably expected that such weather events will contribute to increased financial pressure and uncertainty about the future. In turn, it is likely that distress and suicidality within farming communities will increase [13, 24, 39], unless concerted efforts are made to help buffer these challenges.

Previous research has demonstrated the protective value of social connection for mental health in the general population [40–42], and specifically for farmers [43, 44]. For instance, Woolford et al. [27] reported that Australian farming men relied on their social networks for emotional support, empathy, stress relief, and gaining perspective on their problems. Australian farmers in another study similarly described being able to talk openly with peers about their problems as protective against suicide [45]. In other studies, rural men have expressed a desire for places where they felt comfortable talking about their mental health [46]. Industry-based activities and place-based communities, such as sporting clubs, school parent groups, church groups, or volunteer rural fire services, are important sites of social interaction for farmers [27, 47], and research indicates that these local networks play a crucial role in connecting farmers with health and wellbeing services and providing emotional support during difficult times [47]. This may be related to the trust and credibility that comes with shared identity. Farmers appear to be more willing to seek support from individuals or organisations who they perceive as understanding their way of life [13, 48]. This places professionals who work with farmers in a unique position to offer emotional support and provide relevant information and referrals. Agricultural extension advisors and rural financial counsellors are respected and accessible professionals who are trusted by farmers and who report that they are already offering this kind of support within their current role [49, 50]. Providing spaces where farmers can hear people in their community share their own mental health struggles, and their positive experiences of seeking mental health support, could also help to challenge stigma around help-seeking and normalise conversations of this nature [27].

The importance of involving farmers in the development and delivery of mental health initiatives is becoming more widely recognised [15], and there are a growing number of co-designed wellbeing-focused interventions available to support farmers’ mental health [48, 51–53]. However, reaching and engaging farmers at scale can be difficult, due to pervasive attitudinal barriers that mean they often priortise work over their wellbeing [23], and there is still a need to encourage their uptake of mental health interventions. Holistic, multi-component programs and targeted approaches are needed to address the social, environmental, and cultural factors affecting farmers’ mental health and contributing to their reluctance to access mental health support when needed [15].

There is much evidence to suggest that the social networks we interact with influence our health behaviours [54, 55]. Further evidence supports the effectiveness of online interventions for changing attitudes and behaviours within a social network, using a range of behaviour change mechanisms (e.g., social norms, social support, and social learning) [56, 57]. These approaches can be understood within the context of the socio-ecological model of human behaviour, which conceptualises behavioural outcomes as stemming from interactions between the individual, their closest social circle, the wider community, and society more broadly [58, 59]. According to this model, individuals operate within different social environments (e.g., home, school, workplace, community groups), creating ‘spill over’ effects, where behaviours, attitudes, and beliefs from one social environment can influence another (or from one social network to another). On this basis, previous research has shown that interventions using multi-level approaches to target different social environments are likely to be most effective for changing health behaviours [60]. Social network interventions are often constructed to target different social environments, using specifically selected ‘influence agents’ to intentionally ‘diffuse’ desired behaviours, attitudes, and beliefs within their social networks and across social environments [61, 62]. In their systematic review and meta-analysis, Hunter et al. [55] reported that social network interventions can be effective in changing health behaviours (including behaviours related to wellbeing, alcohol misuse, smoking, and sexual health) in the short and long-term, and within a range of settings and populations. Importantly, they highlighted that social network interventions can be particularly effective in reaching and changing behaviours among ‘hard-to-reach’ populations [55], which farmers can be [52].

Beyond the science, there are obvious benefits to mental health interventions that are based within and targeted to whole communities. For example, involving community members in intervention development and implementation can help to increase their cultural and contextual relevance [63], foster a sense of community ownership and responsibility [64, 65], and enhance community engagement [66]. Moreover, creating space for the expression of local knowledge can build trust with communities, resulting in more effective intervention delivery and improving intervention sustainability [67]. Although a recent review describes a movement towards co-produced community-based mental health initiatives within rural Australia, they note the lack of an evidence base or best-practice guidelines to inform their development [68]. There is also a clear lack of rigorous evaluation, which is surprising given the large amounts of government and philanthropic funding that these types of initiatives attract. For example, in 2022 the Australian Government in partnership with the Foundation for Rural and Regional Renewal provided $4,199,157 to community groups as part of the Future Drought Fund’s Networks to Build Drought Resilience program [69]. Where possible, we believe it is essential for researchers to report on how they have engaged with rural communities to deliver mental health interventions, to build an evidence base and so that interventions may be adapted to other communities. If shown to be effective, key elements can be scaled [68].

We believe that we now need to move beyond mental health awareness raising efforts, to start to meaningfully shift sociocultural norms and therefore overcome attitudinal barriers, equip community members with the skills to talk to each other about these issues, and empower them to engage with evidence-based cognitive, behavioural and/or social strategies that are likely to lead to the maintenance or improvement of their wellbeing. In this paper, we describe the evaluation of a social network campaign designed to increase conversations about mental health and wellbeing, comfort in speaking to others about mental health, and engagement in practical wellbeing-promoting activities in a South Australian farming community.

### Background to the Vocal Locals intervention

#### Local context

The Vocal Locals campaign was based in Loxton, South Australia. Loxton is located 250km north-east of Adelaide and is the central hub of the grain producing Northern Murray Mallee region. The region is one of the driest in South Australia, with an average annual rainfall of 173mm throughout the growing season (April – October) [70]. In 2021, Loxton had a population of 3,947 (52.2% female, median age 47 years), with nearly a quarter (23.8%) of community members in the Loxton-Waikerie local government area employed in agricultural industries [71]. The median weekly household income ($1,093) is significantly lower for Loxton community members than the Australian national median ($1,746).

#### Theoretical underpinning

Based on the socio-ecological model of human behaviour, we designed a social network intervention that operated at multiple levels (i.e., intrapersonal and community) to target different social environments within the Loxton community. Various evidence-based behaviour change techniques (e.g., modelling, education, persuasion) were employed to increase community members’ capability, opportunity, and motivation to perform the target behaviours [72], with a particular focus on modelling and social learning [55, 73, 74].

**Fig. 1:**
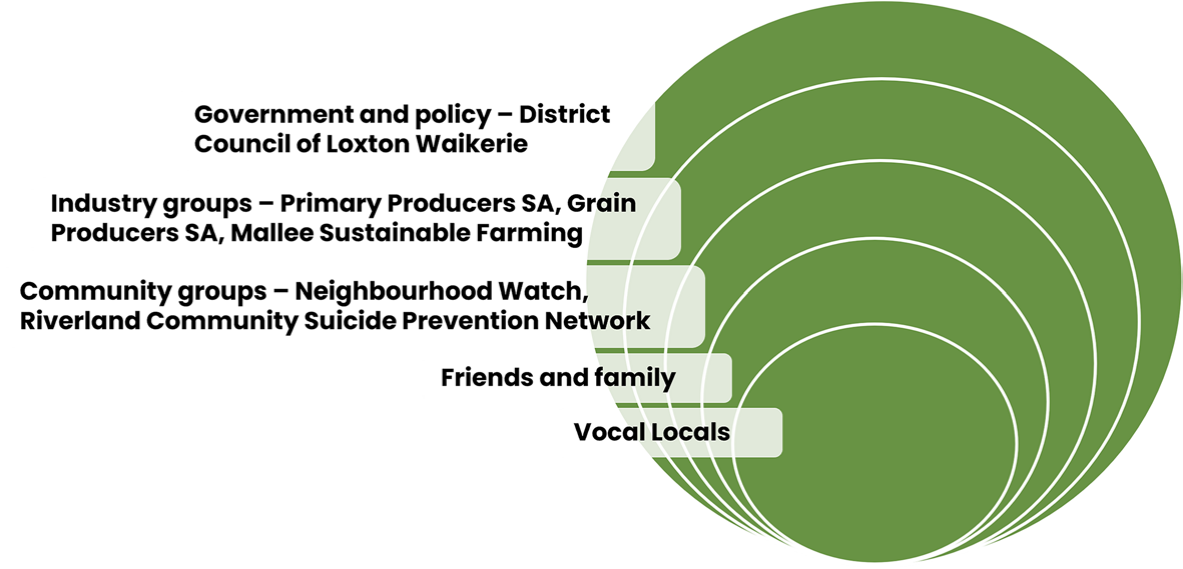
Socio-ecological model of human behaviour applied to the Vocal Locals campaign

We recruited prominent local community members with diverse social networks to become ‘Vocal Locals’ (i.e., ‘influence agents’) and diffuse desired behaviours (e.g., talking about mental health, engaging in wellbeing-promoting activities, supporting others with their mental health) and attitudes (e.g., talking about mental health is okay, engaging in activities for your own wellbeing is important, seeking professional mental health support is smart) within their networks, via modelling and social learning. Vocal Locals were recruited following careful consideration of their social networks and credibility/perceived trustworthiness in the Loxton community. Because the campaign was targeted to farmers and the farming community, we prioritised recruiting local farmers to be Vocal Locals. Figure 1 illustrates the socio-ecological model of human behaviour applied to the Vocal Locals campaign, with five interrelated ‘spheres of influence’ through which desired behaviours and attitudes were diffused. These are *individual* (Vocal Locals), *interpersonal* (friends and family), *community* (e.g., community groups), *institutional or organisational* (e.g., industry groups), and *government and policy*.

Based on recommendations made by Smit and colleagues [75] and in accordance with self-determination theory [76, 77], strategies to enhance Vocal Locals’ intrinsic motivation were built into the intervention design, by promoting autonomy (e.g., flexibility about methods of sharing messages), creating a supportive and respectful group environment (e.g., regular in-person check-ins/social events and a group chat with project team), and equipping them with appropriate training (e.g., completion of ifarmwell modules, social media training, mental health knowledge and skills training) so they felt capable and effective in their roles.

Importantly, the intervention was also designed to target key drivers of poor farmer mental health, including maladaptive sociocultural norms and stigma about help-seeking and investing time in wellbeing-promoting activities [23, 27], low levels of mental health literacy in rural communities [16], use of behavioral disengagement as a coping strategy, and lack of use of acceptance [78], and reduced access to social support and culturally-appropriate mental health support when needed [28, 48]. Further details about the intervention components are described below.

## Methods

### The Vocal Locals intervention

The Vocal Locals campaign is an ifarmwell initiative that aims to promote conversations about wellbeing and challenge attitudes and behaviours that contribute to farmers’ poor mental health. The intervention was designed by the authors (led by KMG) based upon the literature, our previous work with farmers, and through consultation with key stakeholders. More information about the intervention development is provided in Supplementary file 1.

Vocal Locals were purposively recruited through consultation with key stakeholders to ensure that a range of segments of the community were targeted by the intervention. It was important that Vocal Locals met certain selection criteria, including that they were well-respected within their communities, open to talking about mental health and wellbeing, and willing to be in the public sphere. Vocal Locals also needed to be comfortable using social media or willing to learn. Community members who were perceived to meet these criteria were approached in person or by phone by the local project coordinator (JG) and invited to participate in the intervention. Community members who were approached were given clear, detailed information about the intervention and what their participation would involve. One hundred percent of those who were approached agreed to participate.

The intervention was designed to operate at two levels. At an *intrapersonal* level, it aimed to increase Vocal Locals’ knowledge about mental health and wellbeing and strategies that can help to improve it; increase their confidence speaking to others about mental health and supporting people with poor mental health; and improve their wellbeing-related outcomes (i.e., psychological distress and positive mental wellbeing). Intervention components that targeted this were Vocal Locals’ completion of the five ifarmwell Acceptance and Commitment Therapy-based online modules; participation in a half day mental health knowledge and skills training; participation in a half day orientation session; and participation in eight 60-minute wellbeing coaching sessions (in person, via phone, or Zoom). The ifarmwell modules, which are hosted at www.ifarmwell.com.au, were completed by Vocal Locals in their own time and at their own pace. The evidence-based online modules were carefully co-designed with farmers and have been shown to increase positive wellbeing and decrease distress, with benefits maintained at 6-months post-module completion [79]. The mental health training was delivered in person by a local training provider (Mental Health Partners) and was adapted from the Standard Mental Health First Aid. The steering committee advised that the timing of the intervention (a busy period on farms due to seeding, with no flexibility afforded by the funder) meant that it would be unrealistic to ask Vocal Locals to attend a full 2-day training, so a half-day abridged version was delivered instead and recorded for those who could not attend in person. The training focused on improving mental health literacy, reducing stigma, increasing awareness of sources of professional mental health support, and featured someone speaking about their experience living with serious mental illness. This experience effectively modelled to Vocal Locals from the beginning that it is okay to speak about mental health-related topics, and that doing so can help others to feel more comfortable speaking about mental health too. The wellbeing coaching sessions were delivered by a well-respected, local, accredited wellbeing coach with a farming background. Sessions followed a flexible schedule, designed to reinforce and embed learnings from the ifarmwell modules and mental health training, and were underpinned by the GROW model of coaching. This model involves clarification of a challenge or (G)oal, exploration of the current (R)eality, identification of potential (O)ptions, and deciding on next steps or (W)ay forward [80]. As part of their orientation to the campaign, Vocal Locals were provided with a workbook that contained key information about the campaign, a list of local support services, practical tips to improve their wellbeing, a guide to supporting someone who is struggling and/or having thoughts of suicide, and a dedicated space for them to make notes about their learnings. Table 1 contains an overview of the intervention components targeted to Vocal Locals.

**Table 1:** Overview of intervention components targeted to Vocal Locals

if arm well online modules (completed online at own pace):
- Module 1: Taking stock of your current wellbeing and some practical strategies to get you started
- Module 2: Thoughts are like bullies – how to spend less time ‘in your head’
- Module 3: Doing what really matters – how to get the most out of life
- Module 4: Training your ‘attention muscle’ and focusing on the ‘here and now’ – a more pleasant, less exhausting place to be
- Module 5: Putting it all together and moving forward
Mental health knowledge and skills training workshop (half day, adapted from Standard MHFA; delivered in person):
- What is mental health?
- Depression and anxiety – risk factors, signs and symptoms, best practice treatments
- Lived experience perspective
- What can we do to help?
- Self-care
- Local resources
Orientation session (half day; delivered in person):
- Introductions and personal reasons for being a Vocal Local
- Evidence behind the intervention – community-level reasons the Vocal Locals campaign is important
- Evidence behind the intervention – how social network interventions can help
- Using social media to obtain maximum impact – practical tips and having a go
- The mechanics of Vocal Locals – posting on social media and getting the most out of wellbeing coaching
- Reflections and check out
Wellbeing coaching sessions (8 × 60-min sessions; delivered in person, via phone or Zoom):
- Session 1: Getting to know you – current wellbeing, strengths, wellbeing goals
- Session 2: Connecting with the community – clarify why, community connections, and social supports
- Session 3: Managing unhelpful thoughts – stuck thoughts patterns, strategies that help
- Session 4: Doing what really matters – values, aligning behaviours with values
- Session 5: Focusing on the ‘here and now’ – attention, distractions, strategies that help
- Session 6: Putting it all together and moving forward – reflect on learnings, shifts in practice, embedding
- Session 7: Supporting others in the community – how to approach and help a mate, referring to services
- Session 8: Wrapping up – reflect on campaign experiences, intentions going forward
Social events:
- Campaign launch – group meal held after orientation session
- Mid-campaign check-in – debrief with project team, followed by group meal
- Campaign completion – debrief with project team, followed by celebratory meal with family, friends, and campaign supporters

At a *community* level, the purpose of the intervention was to normalise conversations about mental health and wellbeing and increase community members’ engagement in activities that would help them maintain or improve their own wellbeing. Intervention components that targeted this included the opportunity for community members to attend the mental health skills and knowledge training; Vocal Locals sharing what they were doing to improve or maintain their wellbeing and speaking about mental health within their own social networks; and a broader, central communications campaign highlighting Vocal Locals’ wellbeing-related activities and reinforcing key messages (see the Facebook page: www.facebook.com/VocalLocalsIFW/). The broader communications campaign also involved distribution of educational materials (e.g., interviews on local community radio, articles published in local newspapers, a letter box drop to 2,500 households, and notices in local sporting club magazines). The key messages (designed based upon aforementioned research evidence and in consultation with the steering committee) that were repeatedly conveyed as part of the broader communications campaign were:

- Seeking professional mental health help is smart.
- 30 min of exercise has a powerful positive impact on your mental health.
- Sleep is not a luxury – it should be a priority for the sake of your health, mental health, and safety.
- Making time to see your mates is important, no matter how busy life gets.
- Don’t assume that people are okay – ask them!
- For free, practical tips on how to improve your wellbeing, you can visit www.ifarmwell.com.au

Educational materials were distributed via various mediums and included information about local support services, how to support someone who may be having a tough time, and how to support someone who may be suicidal (see Supplementary file 2). Local suicide prevention networks, local government, Members of Parliament, media agencies, and community groups (namely Neighborhood Watch) and sporting leagues assisted with distributing educational materials throughout the community and sharing social media posts on their own platforms. Bumper stickers asking, “What are you doing for your wellbeing today?” and displaying a QR code to www.ifarmwell.com.au were also distributed within the community but were not widely displayed.

The campaign ran from June to August 2022. KMG led discussions with the steering committee and key stakeholders (e.g., community and industry groups, independent contractors, funders) and provided clinical and research oversight of the project, staff and participants. The community-based project coordinator (JG) helped to manage day-to-day activities and supported Vocal Locals throughout the campaign. DW, CMEF, and KMG coordinated the social media and broader communications campaigns, organised campaign events, and liaised with the steering committee and Vocal Locals. CMEF managed activities relating to evaluation of the intervention. Three social events were held throughout the campaign period (i.e., campaign launch, mid-campaign check-in, and at campaign completion) to provide opportunities for Vocal Locals to build group-based identity and cohesion, share experiences, and check-in about how the intervention was progressing. All Vocal Locals and team members were invited to these events (including the wellbeing coach). Vocal Locals were given branded hats and jumpers (sweaters) to further build group-based identity and cultivate a sense of shared meaning and purpose among the Vocal Locals team (acknowledging the importance of group identification for wellbeing; [81]). This also helped to increase Vocal Locals’ visibility within the Loxton community. Photos are included in Supplementary file 2.

The Vocal Locals’ orientation session included training on effective use of social media, and Vocal Locals were strongly encouraged throughout the campaign to use photos and a ‘call-to-action’ at the end of their posts to maximise impact on community members’ behaviour. Social media strategies were based on advice provided by a local agricultural communications business (Ag Communicators) and research examining the use of Twitter for health promotion [82, 83]. For instance, Park et al. [83] found that followers were most likely to engage with tweets that contained action-based messages addressing personal health issues. They also recommend the use of photos and/or videos on social media to enrich message content and highlight the opportunities that social media provides for community-building [83]. Vocal Locals’ social media posts included reflections on how they cope with difficult circumstances and what they do to maintain their wellbeing, and positive stories of reaching out for support and checking in on a friend. Examples of ‘calls-to-action’ used by Vocal Locals were “how do you enjoy connecting with your spouse, friends, or family?”, “when was the last time you gave an old friend a call?”, “what is important to you about being part of a community?”, “what makes you proud?”, “what can you appreciate this week?”, and “where do you go to fill your ‘happiness tank’?”. See more posts on our Facebook page: www.facebook.com/VocalLocalsIFW/.

In addition to their social media posts, Vocal Locals shared information about the campaign and their own wellbeing journeys in their workplaces, sporting groups, farming systems groups, and agricultural bureaus, at regional agricultural field days, and via print and radio media. One Vocal Local, an egg farmer, printed short messages on his eggs to raise mental health awareness – for example, one message was “give it a crack”. Another Vocal Local, a sheep farmer, used a ram auction held on his farm to bring awareness to the campaign. He included a photo of his ram wearing a branded Vocal Locals hat on the front cover of his ram catalogue for the auction and spoke about the campaign to an audience of 80–100 local farmers. Photos are included in Supplementary file 2.

### Evaluation

#### Study design

The impact of the intervention was evaluated via a pre- and post-campaign survey of people who lived in the Northern Murray Mallee region and were familiar with the Vocal Locals campaign, and pre- and post-campaign questionnaires completed by Vocal Locals. The study methods were approved by the University of South Australia Human Research Ethics Committee (protocol number 204624).

#### Procedure

Vocal Locals completed paper-based questionnaires before and after the intervention. Pre- and post-campaign questionnaires included the same measures and were matched using a unique questionnaire code, which helped to maintain confidentiality. Vocal Locals’ demographic characteristics are described in Table 2. Vocal Locals were made aware of the necessarily public nature of their role (i.e., that their participation in the project as a Vocal Local, and the associated research, would be public knowledge) prior to agreeing to participate. Informed consent was indicated by return of signed consent forms upon agreeing to be a Vocal Local. Communication between Vocal Locals and the project team (research team, local project coordinator, and wellbeing coach) was facilitated by a Messenger group chat used throughout the campaign period.

**Table 2:** Demographic characteristics of Vocal Locals

Characteristic	Mean (*SD*)	Number	Percentage (%)
Age (years)	42.8 (12.67)		
Gender			
Woman or female		3	30
Man or male		7	70
Education level			
Did not finish high school		1	10
Finished high school		2	20
Trade certificate, apprenticeship, diploma, or certificate		5	50
Degree or diploma from university		2	20
Occupation			
Farm manager		1	10
Primary producer/Farmer		6	60
Transport operator		1	10
Home duties/Farm partner		1	10
Student		1	10

Community members were invited via social media posts (e.g., District Council of Loxton Waikerie) and emails sent by relevant community groups (e.g., Little Town Productions) to complete an online questionnaire about “an exciting new wellbeing project in Loxton” approximately two weeks prior to the Vocal Locals campaign being launched. Following completion of all intervention activities, a post-campaign questionnaire was distributed by similar channels, plus the newly established Vocal Locals Facebook page. The survey was kept very brief and identifying information was not collected from community members (as recommended by the steering committee to increase engagement and questionnaire completion), therefore pre- and post-questionnaires could not be matched. As per ethical approval, informed consent was indicated by completion of the online questionnaire.

#### Measures

Vocal Locals completed measures of psychological distress (Kessler Psychological Distress Scale [84]) and positive mental wellbeing (Mental Health Continuum-Short Form [85, 86]), as well as measures of general mental health knowledge, stigmatising beliefs about mental illness, and confidence and comfort responding to mental health-related issues, that we adapted from measures previously used to evaluate Mental Health First Aid training [87–89] and successfully used among farmers [90]. Demographic information on age, gender, education level, and occupation was collected as part of the pre-campaign questionnaire.

Community members reported the number of conversations they had about mental health or wellbeing in the last week. These were defined as being *“…about things such as how to manage a difficult situation you or someone else is experiencing, a new activity or hobby that may improve wellbeing, cutting down on bad habits or accessing professional help. If you discussed the same issue with the same person on two different days, this counts as two different conversations”.* They also completed measures of their comfort responding to mental health-related issues and engaging in wellbeing-promoting activities.

The post-campaign questionnaire included questions assessing community members’ familiarity with the Vocal Locals campaign and the impact of the campaign on their own engagement in wellbeing-related activities. Open text response boxes were included to allow community members to describe what they had done differently because of the Vocal Locals campaign and to write any other comments. Postcode and gender were collected at both time points so we could ensure that the sample was comparable. Additional information about these measures is included in Supplementary file 3. Due to time constraints and reporting requirements from the funder, the post-campaign survey was only open for completion for a very brief amount of time (responses collected between 1 September 2022 and 20 September 2022).

#### Statistical analysis

Analysis was conducted using IBM SPSS Statistics version 28. Paired samples t-tests were used to examine changes in Vocal Locals’ levels of psychological distress, positive mental wellbeing, general mental health knowledge, stigmatising beliefs about mental illness, confidence recognising poor mental health, confidence and comfort speaking to others about mental health, and confidence helping someone with poor mental health. Independent samples t-tests were used to examine changes in frequency of conversations about mental health, comfort speaking to others about mental health, comfort engaging in wellbeing-related activities, and self-reported engagement in wellbeing-related activities among community members who lived in the Northern Murray Mallee region and were familiar with the Vocal Locals campaign (i.e., they rated their familiarity as ‘familiar’ or ‘very familiar’ in the post-campaign questionnaire). Responses to open-ended questions included in the post-campaign questionnaire for community members were analysed using content analysis. This method was used because the data collected were short responses to open-ended questions and because it is a descriptive approach that can be used to quantify the frequency with which words, themes, or concepts appear within a dataset [91].

## Results

### Vocals Locals

Vocal Locals’ demographic characteristics are outlined in Table 2. Of the 10 Vocal Locals (70% male; mean age = 42.8 years), eight identified as a primary producer/farmer, farm manager, or farm partner, and they reported working across a range of farm types including grain, livestock (sheep, pigs, poultry, and bees), and horticulture (almonds).

The impact of the Vocal Locals intervention on Vocal Locals themselves was determined by comparing their responses to pre- and post-campaign questionnaires (see Table 3). Paired samples t-tests revealed a significant reduction in psychological distress (*p* = .014), and significant increases in positive mental wellbeing (*p* = .011), general mental health knowledge (*p* = .022), and confidence helping someone with poor mental health (*p* = .004). Increases in confidence (*p* = .066) and comfort (*p* = .081) speaking to others about their mental health approached significance. Reduction in stigmatising beliefs about mental illness (*p* = .102) and increase in confidence recognising poor mental health (*p* = .168) were not significant.

**Table 3:** Paired samples t-test findings comparing Vocal Local’s questionnaire responses pre- and post-campaign

	Pre-campaign (*N* = 10)		Post-campaign (*N* = 10)					
	*M*	*SD*	*M*	*SD*	*df*	*t*	*p*	*Cohen’s d*
Psychological distress (K10)	16.7	3.89	12.7	2.16	9	3.038	**.014**	.961
Positive mental wellbeing (MHC-SF)	56.33	15.72	71.22	7.48	8	-3.299	**.011**	-1.1
General mental health knowledge (single item)	3.11	0.60	3.78	0.44	8	-2.828	**.022**	-.943
Stigmatising beliefs about mental illness (3 items)	1.97	0.68	1.60	0.66	9	1.819	.102	.575
Confidence recognising poor mental health (single item)	3.90	0.32	4.10	0.32	9	-1.500	.168	-.474
Confidence speaking to others about their mental health (single item)	3.30	1.06	4.00	0.00	9	-2.090	.066	-.661
Comfort speaking to others about their mental health (single item)	3.50	0.97	4.10	0.32	9	-1.964	.081	-.621
Confidence helping someone with poor mental health (single item)	2.40	0.69	3.30	0.82	9	-3.857	**.004**	-1.22

In interviews filmed during the final social event and celebration, Vocal Locals reflected on their experiences participating in the campaign. One Vocal Local talked about being recognised by community members: *“…people have seen the Vocal Local sign [logo] on my hat and on the jumper [shirt] I’m wearing … and they’ve asked questions, so yeah I’m just trying to help them out.”* Another stated *“…it’s been a privilege for people to come and just do that to you, tell you a bit about themselves, and you can tell them a bit about yourself, and you can find out you’ve got some things in common.”* The aims of the campaign were highlighted by two Vocal Locals:

…don’t be afraid to be vulnerable, because it’s okay to be vulnerable, and that’s the only way people are going to hear you.

…it might be as simple as smiling, it might be breathing, it might be going outside and enjoying the sunshine and getting some fresh air and a walk. We have resources here, and to not feel like we have to either be struggling so badly that we need to reach out, there are things that we can do right now.

### Community

There were 138 responses to the pre-campaign questionnaire, 65.2% of which came from Loxton, with a further 21% from surrounding districts Berri, Waikerie, and Alawoona. Two-thirds of responses (68.1%) were from women. The post-campaign questionnaire received 152 responses, with 72.4% from Loxton, and a further 17.7% from Berri, Waikerie, and Alawoona. A similar proportion of responses to the post-campaign questionnaire was from women (67.8%). Mean responses to items in the pre- and post-campaign questionnaires are reported in Table 4.

**Table 4:** Independent samples t-tests findings comparing community members’ responses to pre- and post-campaign questionnaires

	Pre-campaign (*n* = 136)		Post-campaign (*n* = 65)^a^					
	Mean (*SD*)	Percentage (%)	Mean (*SD*)	Percentage (%)	*df*	*t*	*p*	*Cohen’s d*
Number of conversations about mental health or wellbeing in week prior	3.26 (3.63)		5.52 (6.85)		82.416	-2.494	**.015**	-.458
Response to item “I am comfortable speaking to others about their mental health or wellbeing”	3.75 (0.99)		4.15 (0.69)		173.232	-3.343	**.001**	-.445
Strongly disagree		3.7		0.0				
Disagree		8.8		0.0				
Neither agree nor disagree		16.2		16.9				
Agree		51.5		50.8				
Strongly agree		19.9		32.3				
Response to item “I am comfortable doing things to maintain or improve my own mental health or wellbeing”	3.93 (0.98)		4.12 (0.82)		199	-1.352	.178	-.204
Strongly disagree		3.7		1.5				
Disagree		5.1		4.6				
Neither agree nor disagree		13.2		4.6				
Agree		50.0		58.5				
Strongly agree		27.9		30.8				
Response to item “In the last week I’ve been doing things to maintain or improve my own mental health or wellbeing”	3.67 (0.98)		4.02 (0.86)		142.848	-2.554	**.012**	-.367
Strongly disagree		2.9		1.5				
Disagree		12.5		4.6				
Neither agree nor disagree		14.7		12.3				
Agree		54.4		53.8				
Strongly agree		15.4		27.7				

^a^t-test conducted using only those living in Northern Murray Mallee region and who reported being ‘familiar’ or ‘very familiar’ with the campaign in the post-campaign questionnaire

Almost half (*n* = 65; 43.9%) of respondents to the post-campaign questionnaire reported being familiar or very familiar with the Vocal Locals campaign. Of those, 58.5% reported that they had done something differently over the previous two months because of the Vocal Locals campaign. These included taking time for themselves, making changes to their mindset, being more aware of their own and others’ mental health, talking more openly about mental health, and having conversations with others about the Vocal Locals campaign (see Table 5).

**Table 5:** Responses from community members about things they had done differently after seeing the Vocal Locals campaign

Category	Frequency^a^	Example response
Taking time for themselves	12	“Taken time to rest instead of pushing through on empty.” (female participant)
		“More purposeful in making time for myself.” (female participant)
Making changes in their mindset or engaging in self-reflection	11	“Thought about how my own actions can benefit or harm my wellbeing and began taking steps to be positive.” (male participant)
		“Thinking differently and seeing positive side more.” (female participant)
Creating new habits or making an effort to do things for wellbeing	6	“Trying to create good habits, and time for me.” (male participant)
		“Making more of an effort to care for my mental health.” (female participant)
Being more aware of their own and others’ mental health	5	“I’ve been more aware of my mental health and ways to improve it.” (female participant)
		“Been more aware of how to help my own mental health, especially while going through a difficult time.” (female participant)
Talking more openly about mental health	5	“Felt comfortable to talk about wellbeing with other people who I may be concerned about.” (female participant)
		“I am more likely to interact with others regarding our wellbeing and mental health.” (female participant)
Getting involved with campaign by talking about it with others or sharing own messages online	5	“It has been a conversation starter with people – ‘have you heard about the Vocal Locals?’ Or ‘I was reading on the Vocal Locals Facebook…’” (male participant)
		“I have had a couple conversations with rural people and directed them to the Vocal Locals Facebook group [page].” (female participant)
Checking in on others	3	“Have taken more time for myself for my own health and wellbeing and have made sure I check in on others more often.” (female participant)
Signing up and using ifarmwell	3	"Registered for the modules.” (female participant)
Engaging in physical activity	2	“More deep conversation, increased walking and yoga, taking time out from work, enjoying the sunshine and little moments of gratitude more.” (female participant)
Supporting others to do things for their wellbeing	1	“Encouraged others who are proactively improving their wellbeing.” (male participant)
Visiting friends	1	“Visited a friend.” (male participant)

^a^Some respondents listed multiple things they had done differently since seeing the campaign, and as a result frequencies are not equal to the number of respondents

The impact of the Vocal Locals intervention within the Loxton community was determined by comparing responses to pre- and post-campaign questionnaires (see Table 4). Independent samples t-tests revealed significant increases in the number of conversations community members were having about mental health or wellbeing (*p* = .015), as well as improvements in comfort speaking to others about their mental health or wellbeing (*p* = .001) and engagement in activities to maintain or improve their own wellbeing (*p* = .012).

Respondents to the post-campaign questionnaire were given the opportunity to provide feedback on the Vocal Locals campaign. Feedback was overwhelmingly positive (see Table 6); of 50 comments provided, 31 (62%) were general positive comments about the campaign. Respondents commented that the Vocal Locals campaign had been an effective way to encourage people to open up and talk about their mental health. One respondent stated that it was a *“great initiative to help those who would not seek help otherwise.”* Another respondent commented: *“I think it has been good to help normalise mental health discussions for some of those who are less comfortable with it. The more they see this info everywhere, the more likely they will be to discuss it.”* Respondents were also keen to see the campaign extended or implemented again. One respondent stated that the campaign *“…should keep going, as the more awareness and ideas for looking after yourself and others, the better!!”* Other respondents thought that although the social media campaign was an effective way to spread wellbeing-related messages, they would have liked more community events to have been held.

**Table 6:** Responses from community members about the Vocal Locals campaign

Category	Frequency	Example response
General positive feedback	31	“It has been a fantastic campaign and a great education tool on the importance of talking about mental health and wellbeing in our communities.” (female participant)
		“Very positive program. Hints shared were simple and achievable.” (female participant)
		“It has been a positive campaign that has great potential to impact local communities raising mental wellbeing awareness.” (male participant)
Positive comment about Vocal Locals	7	“I was having a super bad day [and] [Vocal Local] reached out to me and just said a few things to make my day better. From then on, I have dealt with difficult thoughts in another way.” (female participant)
		“It was everywhere. The people chosen were inspirational and really made you think about yourself.” (male participant)
		“I loved seeing how some of the participants grew in confidence.” (female participant)
Suggestion to run campaign again	6	“A fantastic initiative, we need a second round!” (female participant)
		“Great work. Should keep going, as the more awareness and ideas for looking after yourself and others, the better!!” (female participant)
		“Great initiative, logical to build on the successful pilot.” (male participant)
Other feedback about the campaign	4	“Would have been good to have some community events involving the Vocal Locals rather than just seeing Facebook posts from them” (male participant)
Reflection on increased mental health awareness	2	“Excellent to read the participants hints and experiences and started to notice what I do to help my mental health.” (female participant)

## Discussion

This paper describes the evaluation of the Vocal Locals intervention, a social network campaign designed to improve mental health and wellbeing in Australian farming communities. A strength of the intervention is its contextual appropriateness combined with its theoretical underpinning; it used a multi-level approach grounded in the socio-ecological model of human behaviour, employed evidence-based behaviour change strategies, and built upon the existing strengths of the local community. The resulting impact at both an intrapersonal level (among Vocal Locals) and at a community level (among a sample of community members in the broader Northern Murray Mallee region) is very pleasing, given the well-established challenges associated with engaging farming communities in mental health-focused interventions.

It was not surprising to find that Vocal Locals experienced significant positive outcomes from their participation, given that previous evaluation has shown that completion of the ifarmwell modules reduces distress and improves positive mental wellbeing [79]. The value of wellbeing coaching in helping people to pursue personal goals [92–95] and the importance of social connection for wellbeing [40–42] is also demonstrated in the literature. However, creating change at a community level is typically far more difficult. We aimed to develop an intervention that would move beyond raising awareness of mental health issues and begin to shift sociocultural norms, overcome attitudinal barriers, equip community members with the skills to talk openly about mental health, and empower them to engage in strategies to maintain or improve their wellbeing. Our evaluation shows promise, with significant shifts found in the number of conversations a sample of community members were having about mental health and wellbeing, their level of comfort speaking to others about their mental health and wellbeing, and their reported engagement in wellbeing-related activities to maintain or improve their own mental health and wellbeing.

To our knowledge, this is the first initiative using a social network approach to specifically focus on improving mental health and wellbeing in an Australian farming community. Similar wellbeing initiatives tend to be implemented over a longer term, often with on-going evaluation. For example, ‘Our Healthy Clarence’, established in the Clarence Valley in Northern New South Wales, involved community-driven development and implementation of a mental health and wellbeing plan over a two-year period [96]. An evaluation was conducted two years after the project was established and involved review of project documents and quantitative information (e.g., numbers of attendees to training events) and semi-structured interviews conducted with key stakeholders (e.g., steering committee members, community members, volunteers, and service providers) [96]. The evaluation demonstrated the impact of the initiative in empowering community members and shifting narratives about mental health and wellbeing in the Clarence Valley [96]. Another wellbeing initiative, ‘Act-Belong-Commit’, was first piloted in regional communities and has been running across Western Australia since 2008 [97]. This initiative is a state-wide mass media campaign that aims to reframe perceptions of mental health as something that can be proactively protected and strengthened, rather than simply being the absence of mental illness [97]. The impact of the campaign has been evaluated via telephone interviews conducted with the general population and various subgroups, such as school students. Cross-sectional findings suggest that the campaign is effective in reducing stigma surrounding mental illness, encouraging conversations about mental health issues, and improving engagement with wellbeing-promoting activities [98], including mental health help-seeking [99]. In contrast to these much larger initiatives, the Vocal Locals campaign was a short-term project, running over a two-month period. Despite this limitation, we were able to conduct pre-/post- survey of Vocal Locals and broader community members to evaluate the impact of the campaign, with significant shifts in target outcomes.

Although we did not evaluate this specifically, our findings indicate the potential benefits of the intervention in challenging stigma and attitudinal barriers and increasing help-seeking behaviours among community members, reflected by increased comfort talking with others about mental health and wellbeing. Due to time and resource constraints, we were not able to examine the impact of the Vocal Locals campaign on mental health service uptake in the region. As highlighted by De Cotta and colleagues [68], identifying changes in mental health service use can be difficult as outcomes take time to realise. This is also the case for identifying changes in suicide rates; although, as Powell et al. [96] highlighted, there are additional difficulties attributing such changes directly to wellbeing initiatives with broader social factors at play. In general, researchers have struggled to link gatekeeper training and public education to reductions in suicide rates [100, 101]. Also, while obviously very alarming, suicides remain a relatively rare occurrence, even in rural communities. It is worth noting that almost half of the new registrations on www.ifarmwell.com.au during the study period were from the Northern Murray Mallee region, pointing to a positive impact of the campaign on local farmers investing time in their wellbeing and their uptake of support services. This is significant, given research showing that farmers are half as likely to have seen a GP or mental health professional in the previous 12 months than other rural people [102]. More work is urgently needed across Australia, to challenge attitudes, shift sociocultural norms, and increase help-seeking among this at-risk group.

There are several factors that we believe were important in the success of the Vocal Locals campaign. First, the ifarmwell modules are freely available and were carefully co-designed with farmers to combine their preferences for online interventions with evidence-based therapeutic approaches (e.g., Acceptance and Commitment Therapy) that target the particular challenges farmers are known to face [48, 51, 79]. Second, Vocal Locals were provided with a supportive environment where they were given the opportunity to learn practical skills and strategies to manage their own mental health and wellbeing (e.g., via the ifarmwell modules, wellbeing coaching sessions, group social events and group chat), in addition to supporting others with poor mental health (e.g., via mental health knowledge and skills training). Each component of the intervention was designed to build Vocal Locals’ intrinsic motivation by fostering feelings of competence (capability and effectiveness), relatedness (being respected and supported by others), and autonomy (having choice and responsibility) using self-determination theory-based techniques [75–77]. Fostering group collaboration and a sense of belonging among Vocal Locals throughout the campaign appeared crucial and, anecdotally, was highly valued by participants. It should be noted that some concerns about the logistics of the campaign were raised by Vocal Locals during the mid-campaign debrief (e.g., frequency of coaching sessions, processes for posting and sharing social media posts), and efforts were made to quickly accommodate their suggestions and requests. Third, educational materials that were distributed as part of the broader communications campaign included information about local support services and how to support someone who may be struggling and/or having thoughts of suicide. This was an important inclusion because rather than general awareness raising, community members were provided with information on actions they could take to support others, including information on where they could refer people within their local region. Upskilling community members in this way challenges negative attitudes and stigma and helps to ensure timely access to knowledge and support (including social support within the community), given that services in rural regions may be fragmented or inaccessible when needed [103]. We also believe that reinforcing our five key messages via multiple mediums helped ensure these messages were heard, remembered, and acted upon.

The success of the Vocal Locals campaign must be considered in light of potential limitations. We were significantly limited by resource and time constraints in our design and implementation of the campaign in Loxton. This meant that we were also unable to engage in detailed co-design work with a broad range of community members, as is widely recognised as being integral for better service design and uptake [68] and is our strong preference. We discuss how we would have involved the community in a more in-depth co-design process, given additional resources and time, in Supplementary file 1. To address this limitation, we embedded community members and leaders as part of our project team and steering committee and remained as open and flexible to Vocal Locals’ suggestions as the timeframe and budget allowed. Qualitative feedback from community members indicated a desire for more community events, and this may be an area for adaptation or expansion should the Vocal Locals campaign be run in other communities. However, it is worth noting that we did not want to duplicate the work of other groups (e.g., Suicide Prevention Networks and Mental Health First Aid). Instead, our aim was to complement other services and encourage uptake of mental health and wellbeing-related activities already present in the region.

We also wish to acknowledge limitations of our evaluation. Firstly, the pre-/post- survey of the broader community did not use repeated measures or matched responses, reducing the power of the evaluation. The absence of a control community similarly reduces the confidence with which shifts in target outcomes can be attributed solely to the campaign. Secondly, the number of community members who responded to the questionnaire at each timepoint was quite small, likely as a result of the brief windows in which we had to conduct these surveys due to the funding agreement. In the interests of minimising participant burden, minimal demographic data was collected, but this meant we could not assess whether the sample was representative of the Loxton community. This also makes it difficult to know whether the campaign was reaching the target audience (farmers), given that information about occupation was not collected. Demographic information about cultural background, including Aboriginal and Torres Strait Islander status, was also not collected. Finally, less than half (*n* = 65; 43.9%) of those who responded to the post-campaign questionnaire reported being familiar or very familiar with the Vocal Locals campaign, which raises further questions about its reach. These are important to consider while also recognising that the Vocal Locals campaign was a short-term project, running over a two-month period. Where possible, future trials of the Vocal Locals campaign should be evaluated using more rigorous study designs.

The Vocal Locals campaign could be replicated in farming communities across Australia, with adaptations for the local context, and our next step is to finalise a protocol describing step-by-step actions to implement the Vocal Locals intervention (this will be published on our website: www.ifarmwell.com.au). It is unclear whether this intervention would be appropriate across all rural areas and in communities where there are other key economic drivers (e.g., mining or fishing-focused communities). We designed the campaign with farmers, and for a farming community, based on our extensive knowledge of this population. We believe this model is likely to work best in smaller communities, where there are shared identities, high levels of trust, well-established social connections, and non-transient populations. Similarly, it is difficult to say whether a model like this would be appropriate for Aboriginal and Torres Strait Islander communities. However, kinship and family structures and strong cultural values may support implementation of an intervention based on this model, with appropriate adaptation for Aboriginal communities and their specific context.

It is important to note that a basic premise of the socio-ecological model of human behaviour is that educating, upskilling, and motivating individuals to engage in health-promoting behaviours is difficult or impossible without also creating environments in which it is convenient, socially desirable, and economical to engage in these behaviours [60]. In support of this, Farmer and colleagues [103] highlight the need for national advocacy and a national movement to address rural mental health. This aligns with our calls for national efforts to promote broader cultural shifts of dominant farming cultural ideals that compromise farmers’ wellbeing (e.g., overly valuing productivity at the cost of wellbeing) [104]. Given the success of the Vocal Locals intervention, and that negative attitudes towards mental illness, mental health professionals, and help-seeking exist throughout the agricultural industry across Australia [23, 26, 27], we believe a useful next step would be to test whether a similar national ambassador campaign, implemented over a longer period and using a broader range of mediums (e.g., regional television advertisements), could shift attitudes in farming communities more broadly, and therefore create environments where wellbeing-promoting behaviours are convenient and more socially desirable for farmers to engage with. This is our ultimate goal given the elevated rates of suicide and lost productivity and social cohesion resulting from unmanaged poor wellbeing in this group.

## Conclusions

The Vocal Locals campaign brought together expertise from science and community members to achieve meaningful outcomes for an Australian farming community. Evaluation of the campaign in Loxton demonstrated significant positive outcomes for individuals who participated as Vocal Locals and for respondents to a community survey who were familiar with the campaign. The Vocal Locals campaign may serve as a model for others who wish to challenge sociocultural norms and attitudinal barriers to help-seeking, normalise conversations about mental health and wellbeing, and empower those in farming communities to engage in strategies to maintain or improve their wellbeing.

### Supplementary Information

**Supplementary Material 1.**

**Supplementary Material 2.**

**Supplementary Material 3.**

## Data Availability

The datasets generated and analysed during the current study are not publicly available to maintain confidentiality of the Vocal Locals who participated. The community dataset is available from the corresponding author on reasonable request.
