# Supplementary material for: A ‘Vocal Locals’ social network campaign is associated with increased frequency of conversations about mental health and improved engagement in wellbeing-promoting activities in an Australian farming community
Source: BMC Public Health. 2024 Mar 2;24:673. doi: 10.1186/s12889-024-18193-7 (PMC10909292; doi:10.1186/s12889-024-18193-7)
Supplement: Supplementary file 1 — Supplementary Material 1. [file 12889_2024_18193_MOESM1_ESM.docx]

**Supplementary File 1: Additional details on intervention development**

1. ***Consultation with stakeholders***

The Vocal Locals campaign was designed by the authors (led by KMG) based upon the literature, our previous work with farmers, and through consultation with key stakeholders. A steering committee (*n*=9) was formed made up of agricultural industry bodies (Chief Executive Officers, Primary Producers SA and Grain Producers SA), a local farming systems group (Project Manager, Mallee Sustainable Farming), representatives from the local council (Councillor and Director of Commercial and Community Services, District Council of Loxton Waikerie), agricultural communications professionals (Managing Director and Project Manager Ag Communicators), and members of the farming community in the Northern Murray Mallee region of South Australia. The steering committee informed the design of the intervention (e.g., intervention components, campaign name, key messages) and the Vocal Locals role (e.g., purpose, role description, desired qualities), and were consulted on selecting Vocal Locals from the community. Members of the steering committee were also involved in implementing the broader communications campaign (e.g., by sharing posts on social media and advertising the mental health training in the community). It is important to note that, because the campaign was targeted to members of the farming community, the steering committee was composed primarily of representatives from agricultural-focused groups.

1. ***Vocal Locals selection***

Ten prominent local community members were recruited by the local project coordinator (JG) to become ‘Vocal Locals’ and act as advocates for mental health and wellbeing within their social networks. When selecting Vocal Locals, we considered how various perspectives and segments of the community could be represented, so that the campaign could reach as many community members as possible. Because the campaign was targeted to farmers and the farming community, we prioritised recruiting local farmers to be Vocal Locals. We also recruited non-farming community members and ensured that there was diversity in age, gender, and (for farmers) farm type. In the final group, there were eight farmers.

1. ***Reflections on co-design***

Due to the very tight timeframe imposed by the funder (i.e., the funding period was from March to end of August 2022 and including seeding time which meant farmers and farming groups could not engage with us during much of this period), we did not have time to perform the level of co-design work we would have like to, and that we have with all of our previous work with farmers and rural communities (e.g., 1, 2-4). Despite this challenge, we were able to draw upon broad research, clinical, and life experience from within the project team, in addition to consulting with the steering committee. Three of the four authors (KMG, DW, and CMEF) have farming and/or rural backgrounds, and this was helped by having a local farmer (JG) work as the local project coordinator and a key member of the project team. This helped us to respond proactively to feedback from industry groups and Vocal Locals.

Given time and resources, we would have engaged in a more in-depth co-design process with the community. Our previous work has drawn upon existing co-design frameworks (e.g., the seven-step framework developed by Trischler and colleagues (5)), guided by four key methodological approaches: (1) synthesising evidence from prior research to understand the problem and possible solutions, (2) using intervention mapping techniques to chart intervention logic (e.g., relevant behaviour change strategies), (3) using a person-centred approach through the involvement of farmers as co-designers, and (4) making collaborative and iterative changes to the intervention based on participant feedback (4). In practice, this would have involved conducting interviews or focus groups with local farmers and community members to gain insight to wellbeing-related challenges that are specific to their community and to explore how they would like to be supported to overcome these challenges. The community would have been involved in co-designing various aspects of the intervention, for example by nominating community members to be Vocal Locals, providing feedback on key campaign messages, and organising campaign activities. Involving the community in this way would have helped to ensure that the intervention was appropriate for their context, addressed their concerns, and met their needs and preferences. Also, when participants are involved in co-design or participatory action research, they often move from being participants to being advocates for the initiative that they are helping to develop or implement. This was observed by authors of ‘Our Healthy Clarence’, who reported that involving community members in designing and implementing the initiative empowered them to address their concerns as they arose (6). Vocal Locals would have similarly been involved in co-designing their role, the logistics of the campaign (e.g., method of sharing campaign messages), educational materials, and other campaign activities. It should be noted that although Vocal Locals were not strictly involved as co-designers, we were able to make iterative changes to the intervention based on their feedback. For instance, some concerns about the logistics of the campaign were raised by Vocal Locals during the mid-campaign debrief (e.g., frequency of wellbeing coaching sessions), and we were able to quickly accommodate their suggestions and requests. It should also be noted that the Vocal Locals campaign was found to be widely acceptable and impactful through our evaluation. Therefore, we hope that by drawing upon our previous experience working in this space, and key members of the team having much relevant lived experience, we were able to minimise the negative impact of not engaging in detailed co-design processes (that resulted from timing challenges imposed by the funder).

**References**

1. Gunn KM, Barrett A, Hughes-Barton D, Turnbull D, Short CE, Brumby S, Skaczkowski G, Dollam J. What farmers want from mental health and wellbeing-focused websites and online interventions. J Rural Stud. 2021;86:298-308. <https://doi.org/10.1016/j.jrurstud.2021.06.016>

2. Gunn KM, Skaczkowski G, Dollman J, Vincent AD, Short CE, Brumby S, Barrett A, Harrison N, Turnbull D. Combining farmers' preferences with evidence-based strategies to prevent and lower farmers' distress: Co-design and acceptability testing of ifarmwell. JMIR Hum Factors. 2022;9(1):e27631. <https://doi.org/10.2196/27631>

3. Fennell KM, Turnbull DA, Bidargaddi N, McWha JL, Davies M, Olver I. The consumer-driven development and acceptability testing of a website designed to connect rural cancer patients and their families, carers and health professionals with appropriate information and psychosocial support. Eur J Cancer Care. 2017;26(5):e12533. <https://doi.org/10.1111/ecc.12533>

4. Kennedy A, Cosgrave C, Macdonald J, Gunn K, Dietrich T, Brumby S. Translating co-design from face-to-face to online: An Australian primary producer project conducted during COVID-19. Int J Environ Res Public Health. 2021;18(8). <https://doi.org/10.3390%2Fijerph18084147>

5. Trischler J, Dietrich T, Rundle-Thiele S. Co-design: From expert-to user-driven ideas in public service design. Public Management Review. 2019;21(11):1595-619. <https://doi.org/10.1080/14719037.2019.1619810>

6. Powell N, Dalton H, Perkins D, Considine R, Hughes S, Osborne S, Buss R. Our Healthy Clarence: A community-driven wellbeing initiative. Int J Environ Res Public Health. 2019;16(19):3691. <https://doi.org/10.3390/ijerph16193691>
