# Supplementary material for: A ‘Vocal Locals’ social network campaign is associated with increased frequency of conversations about mental health and improved engagement in wellbeing-promoting activities in an Australian farming community
Source: BMC Public Health. 2024 Mar 2;24:673. doi: 10.1186/s12889-024-18193-7 (PMC10909292; doi:10.1186/s12889-024-18193-7)

**Supplementary File 2: Pictures documenting the campaign**

**Figure S1**

*One of the educational flyers distributed as part of the broader, central communications campaign.*


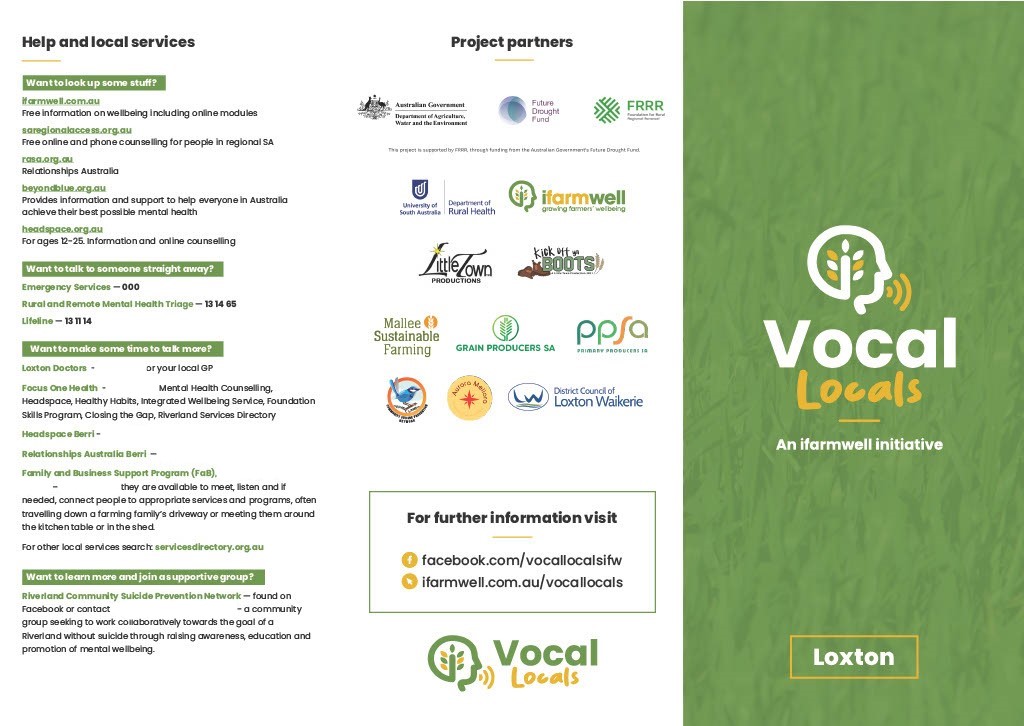

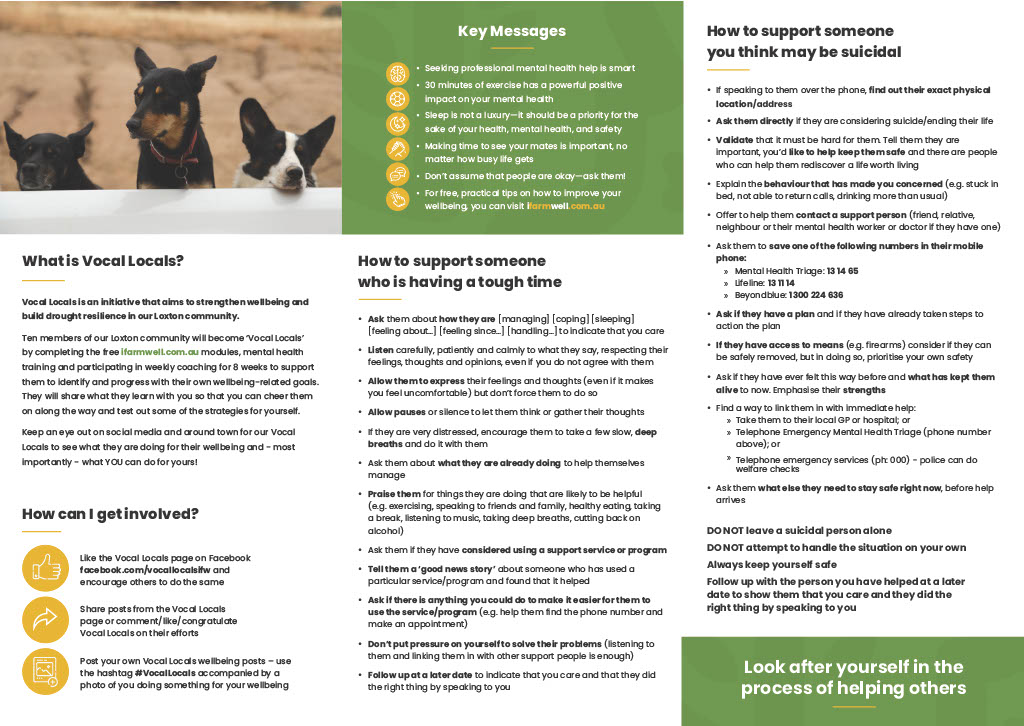


**Figure S2**

*Branded hats and shirts were given to Vocal Locals to facilitate development of a group-based identity and cultivate a sense of shared meaning and purpose. The top picture shows the project team modelling the branded shirt. The bottom picture was taken by a Vocal Locals and shows the branded hat.*


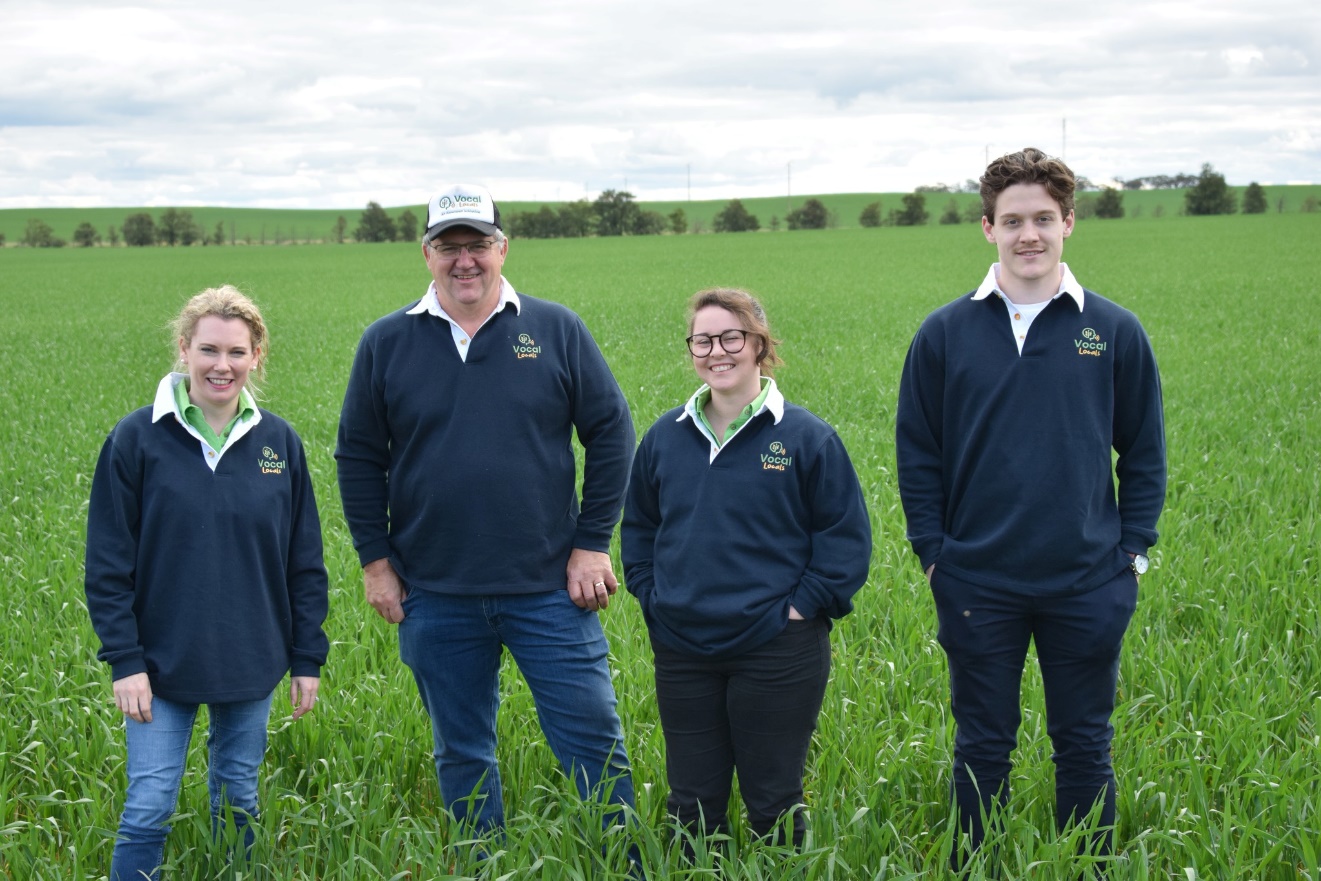

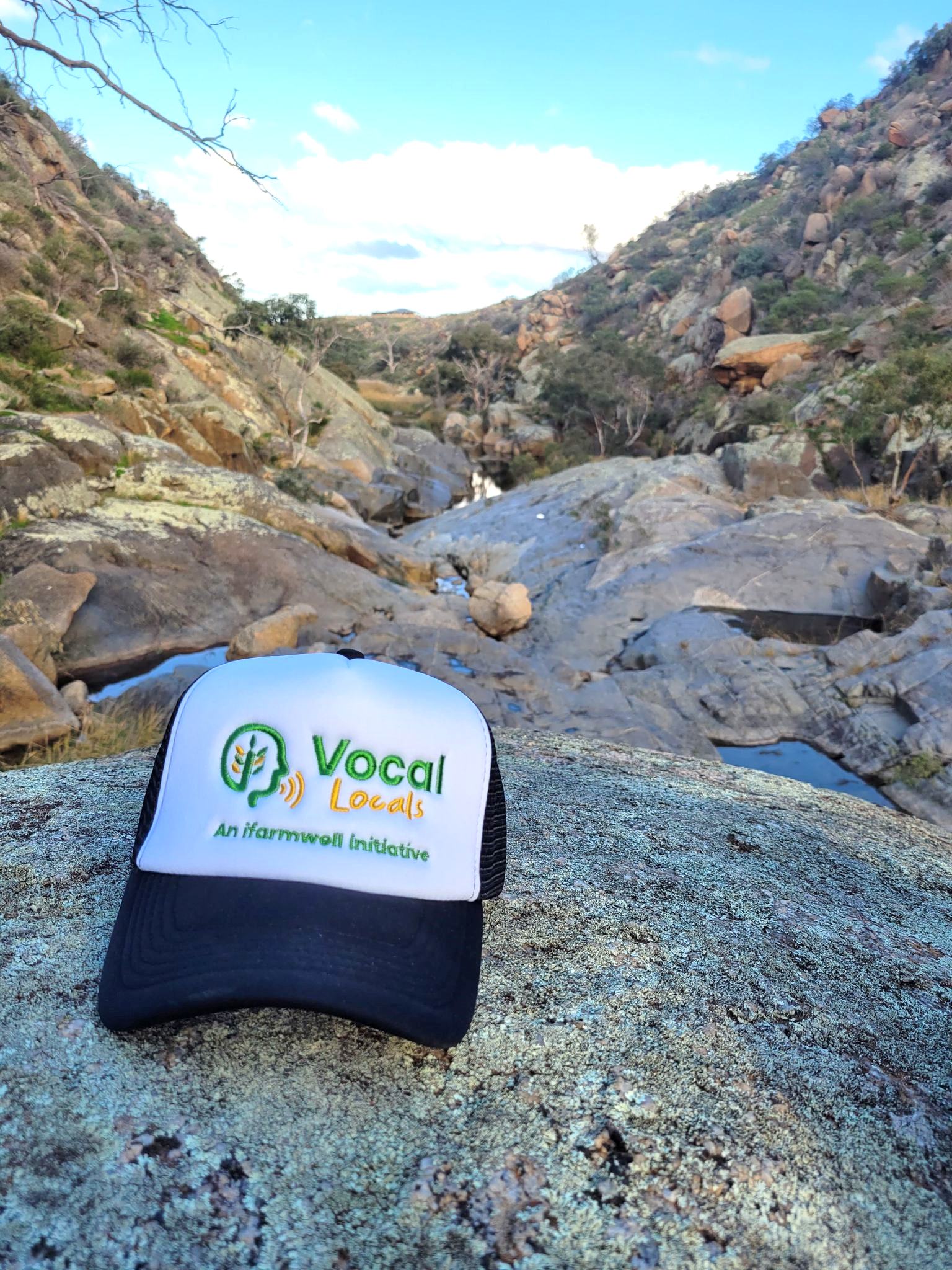


**Figure S3**

*Example social media post written by a Vocal Local and posted on central campaign page.*


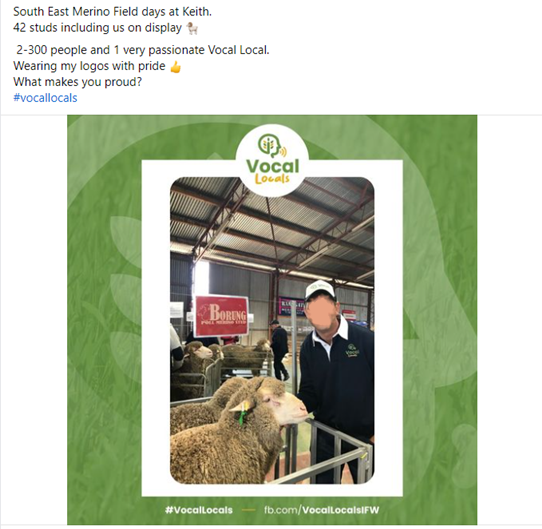


**Figure S4**

*Example social media post written by a Vocal Local and posted on central campaign page.*


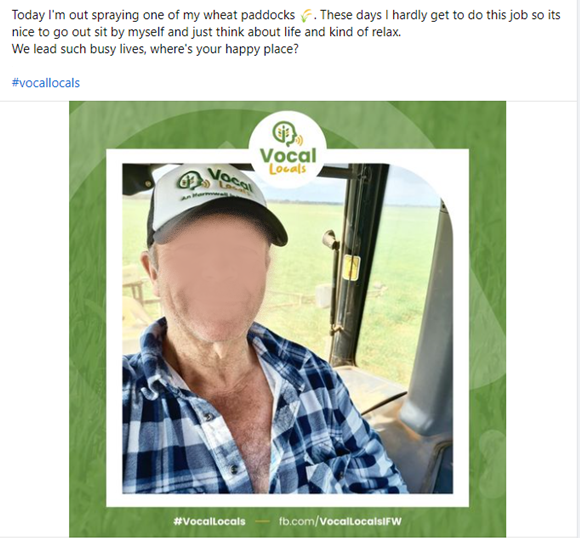


**Figure S5**

*Example social media post written by a Vocal Local and posted on central campaign page.*


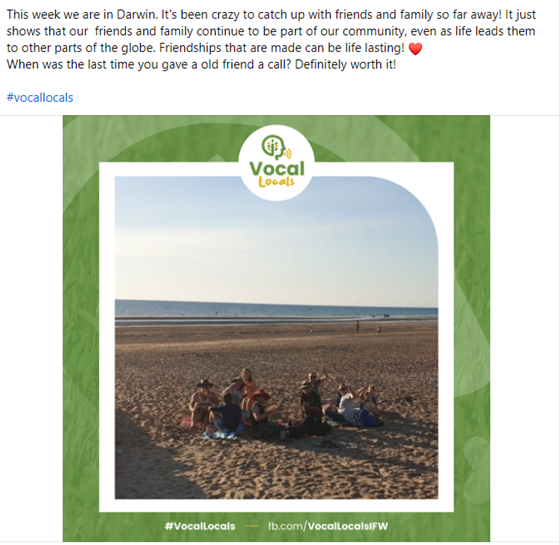


**Figure S6**

*Example social media post written and posted by a Vocal Local.*

**
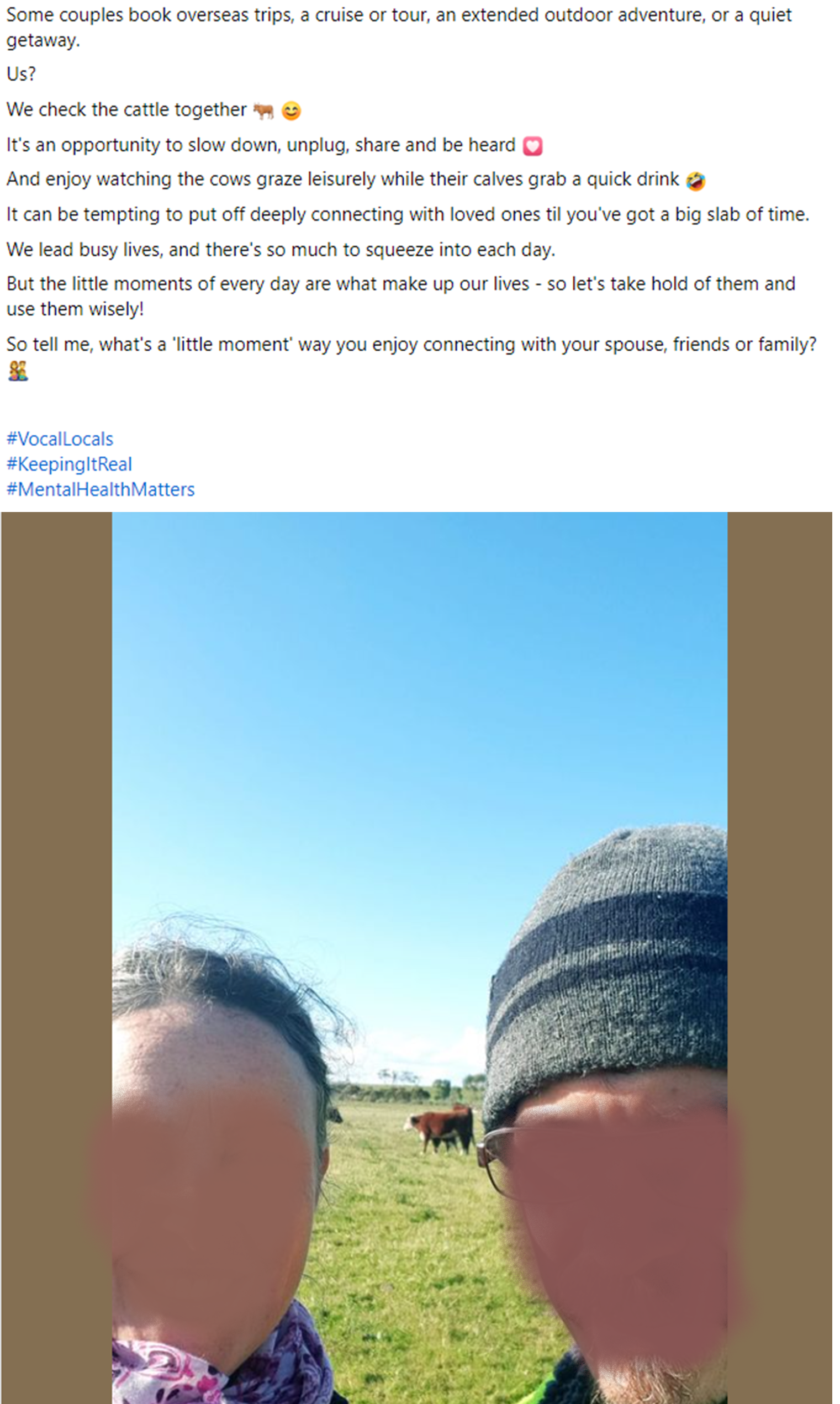
**

**Figure S7**

*Example social media post written and posted by a Vocal Local.*

**
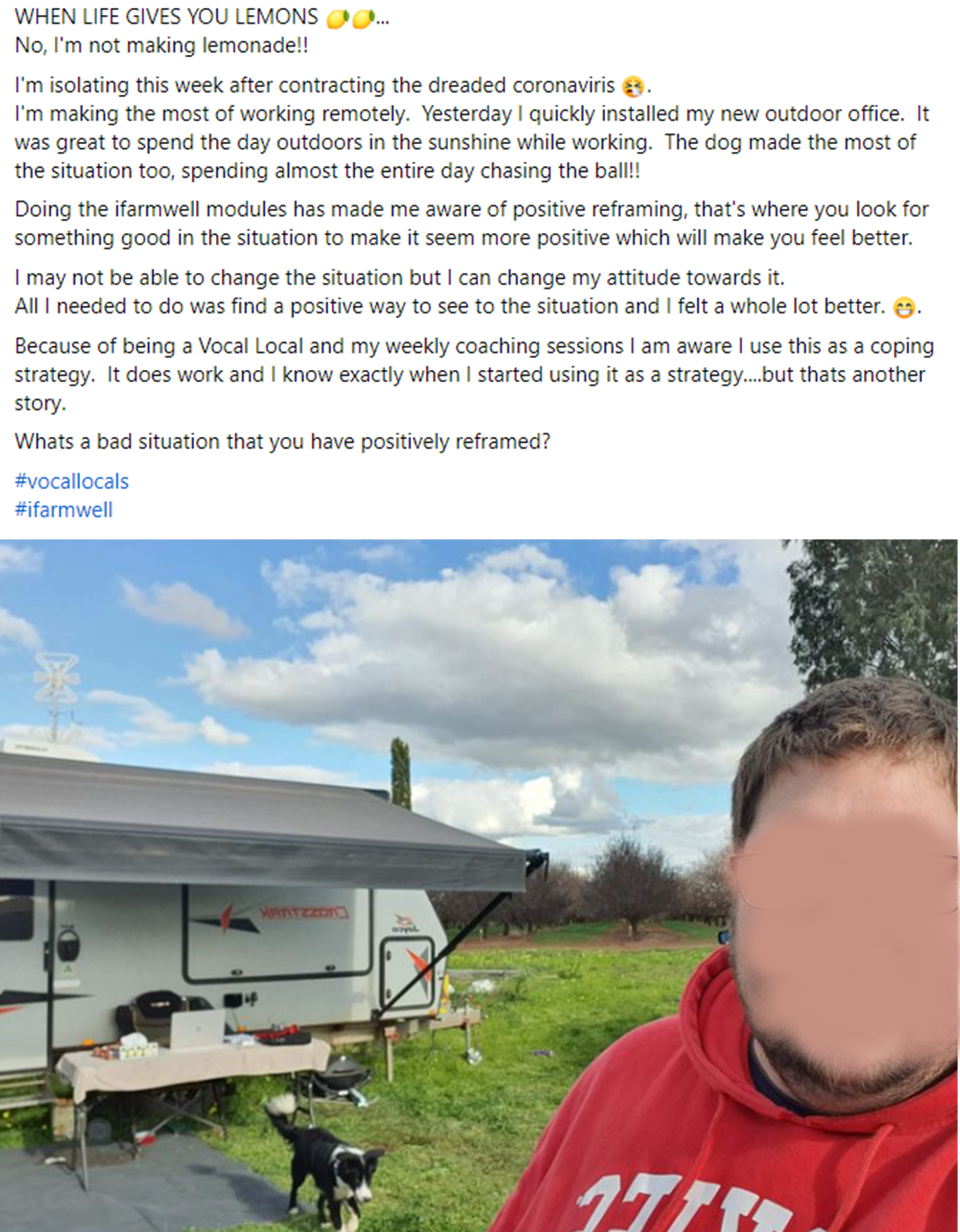
**

**Figure S8**


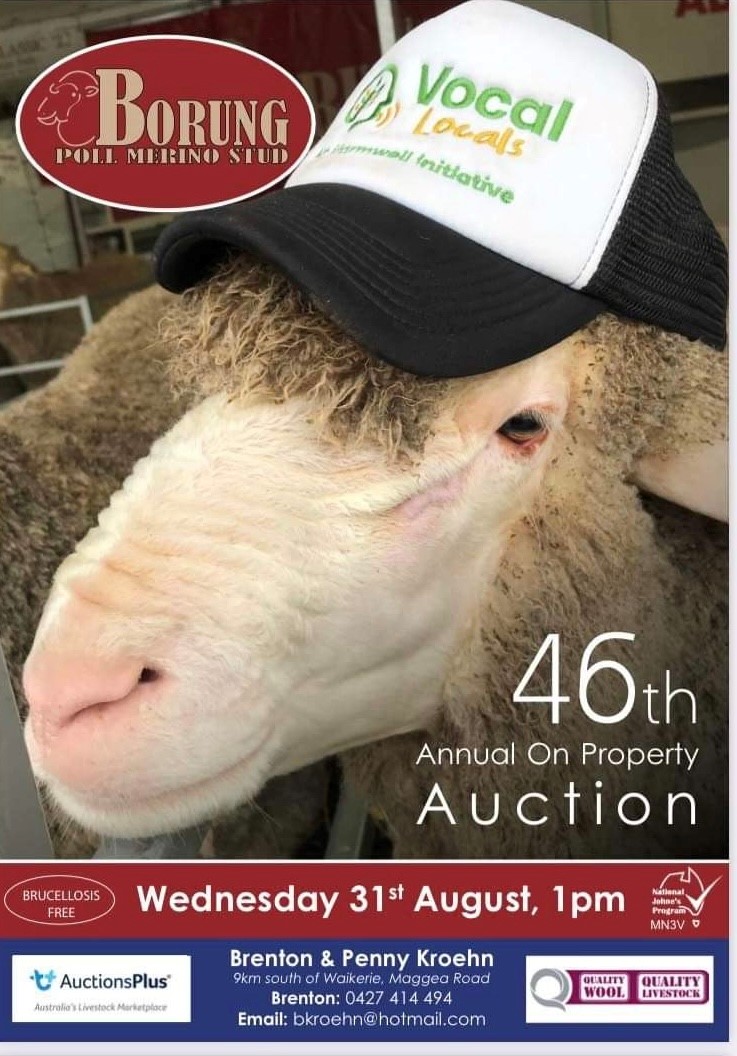
*One Vocal Local, a sheep farmer, included a photo of his ram wearing a branded hat on the front cover of his ram catalogue for an auction held on his farm.*

**Figure S9**

*One Vocal Local, an egg farmer, printed short messages printed on his eggs to raise mental health awareness.*


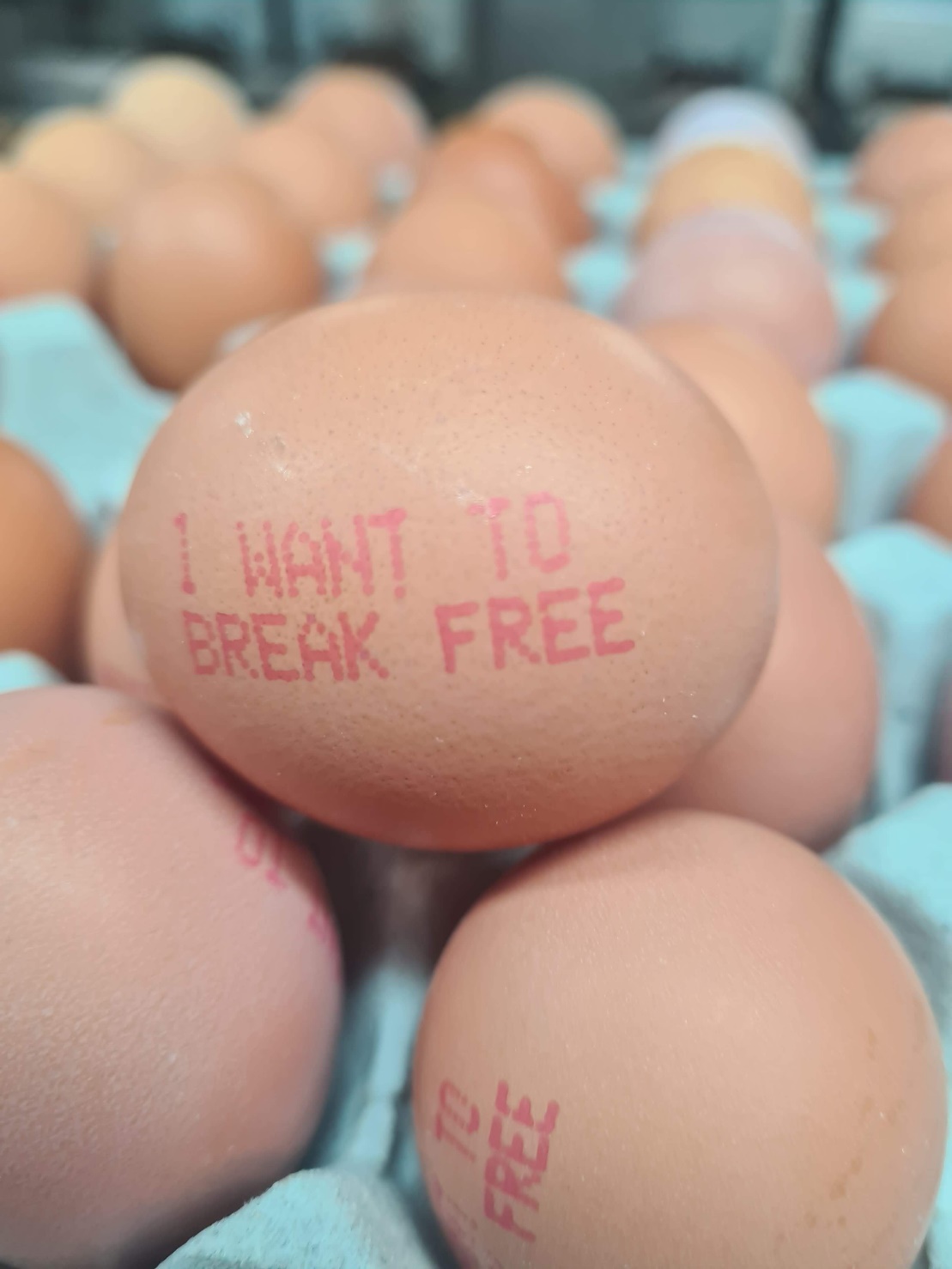

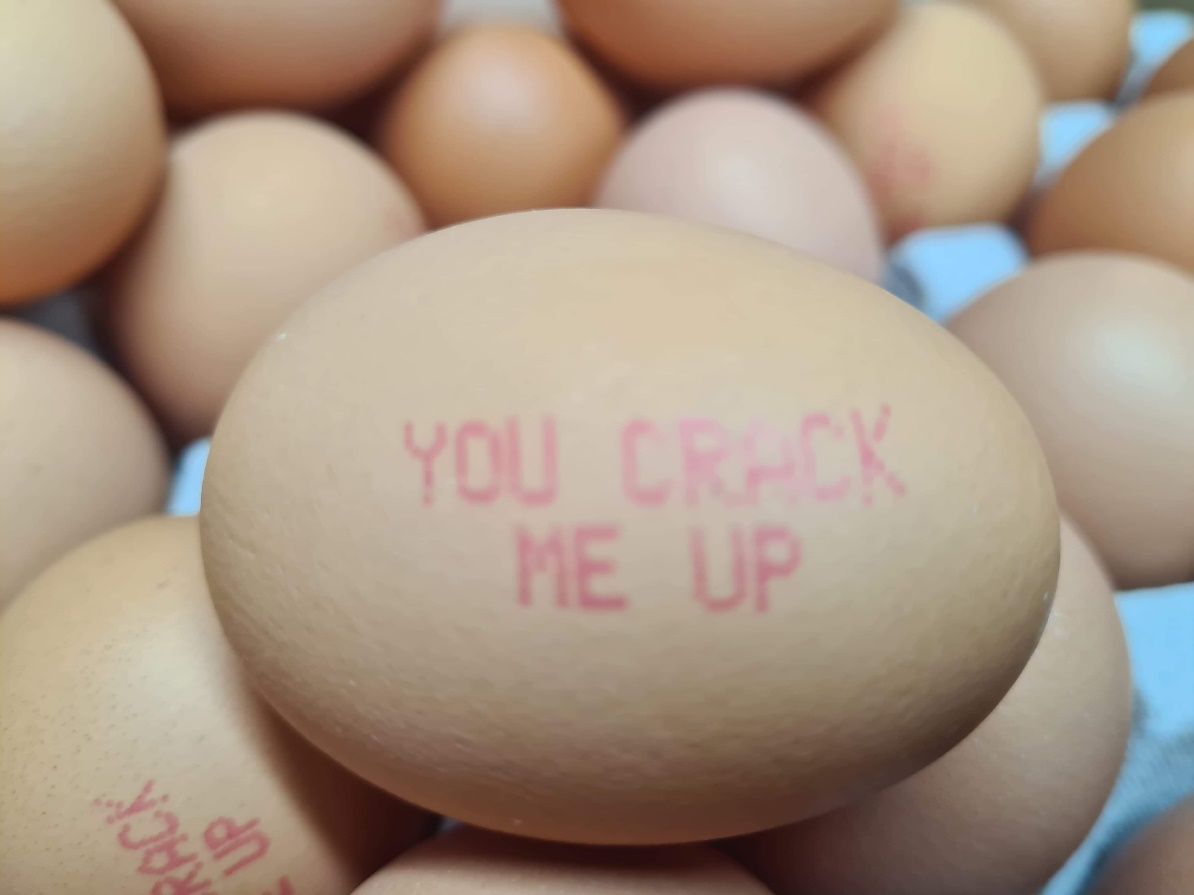

Supplement: Supplementary file 2 — Supplementary Material 2. [file 12889_2024_18193_MOESM2_ESM.docx]
