# Supplementary material for: A ‘Vocal Locals’ social network campaign is associated with increased frequency of conversations about mental health and improved engagement in wellbeing-promoting activities in an Australian farming community
Source: BMC Public Health. 2024 Mar 2;24:673. doi: 10.1186/s12889-024-18193-7 (PMC10909292; doi:10.1186/s12889-024-18193-7)
Supplement: Supplementary file 3 — Supplementary Material 3. [file 12889_2024_18193_MOESM3_ESM.docx]

**Supplementary File 3: Additional details on measures used in evaluation**

1. ***Measures included in Vocal Locals questionnaires***

Vocal Locals completed paper-based questionnaires before and after the intervention. Pre- and post-campaign questionnaires included the same measures and were matched using a unique questionnaire code. Each questionnaire included measures of psychological distress, positive mental wellbeing, general mental health knowledge, stigmatising beliefs about mental illness, and confidence and comfort responding to mental health-related issues. Measures used are described in detail below.

Psychological distress was assessed using the 10-item Kessler Psychological Distress Scale (K10) (1). Vocal Locals were asked to reflect on how they had been feeling during the previous 30 days and respond to items using a five-point Likert scale (1 = None of the time, 2 = A little of the time, 3 = Some of the time, 4 = Most of the time, 5 = All of the time). Total scores range from 10 to 50, with higher scores indicating greater distress. A continuous score can be used in analyses or participants can be classified as having low (10–19), mild (20–24), moderate (25– 29), or severe (30–50) levels of distress.

Positive mental wellbeing was assessed using the 14-item Mental Health Continuum-Short Form (MHC-SF) (2). Vocal Locals responded to items according to how they had felt during the previous 30 days. Responses were indicated on a six-point Likert scale, where 0 = Never and 5 = Every day. Total scores range from 0 to 70, with higher scores indicating better mental wellbeing. The scale can be scored as a continuous measure or to categorically indicate “languishing”, “moderate” or “flourishing” wellbeing (3).

Measures of general mental health knowledge, stigmatising beliefs about mental illness, and confidence and comfort responding to mental health-related issues were adapted from measures previously used to evaluate Mental Health First Aid training (4-6) and successfully used among farmers (7).

More specifically, general mental health knowledge was assessed using a single item (“*how would you rate your current knowledge about general mental health concerns, e.g., depression, anxiety, inability to concentrate?”*) which was rated on a five-point Likert scale, where 1 = I have no knowledge about these concerns and 5 = I am very knowledgeable about these concerns.

Stigmatising beliefs about mental illness were assessed using three items (*“I believe that farmers with depression could snap out of it if they wanted*”, *“I believe that farmers with depression or thoughts of suicide could snap out of it if they wanted”*, and *“I believe that farmers who say they are stressed are complaining too much”*) rated on a five-point Likert scale from 1 = Strongly disagree to 5 = Strongly agree.

Confidence recognising poor mental health was assessed using a single item (“*I am confident that I could recognise when someone is struggling with their mental health or wellbeing”*). Confidence and comfort speaking to others about their mental health were assessed using two single items (*“if someone approached me to have a conversation about their mental health or wellbeing, I am confident I would know how to respond in an appropriate way”* and *“I am comfortable speaking to others about their mental health or wellbeing”*, respectively). Each of these items were rated on five-point Likert scales from 1 = Strongly disagree to 5 = Strongly agree.

Confidence helping someone with poor mental health was assessed using a single item (*“how confident would you feel in helping someone with poor mental health?”*) which was rated on a five-point Likert scale, where 1 = Not at all confident and 5 = Very confident.

Demographic information on age, gender, education level, and occupation was collected as part of the pre-campaign questionnaire.

1. ***Measures included in community questionnaires***

Community members were surveyed before and after the intervention. Pre- and post-campaign questionnaires included the same measures, except for additional questions that were included in the post-campaign questionnaire to ask specifically about the impact of the Vocal Locals campaign.

Participants reported the number of conversations they had about mental health or wellbeing in the last week. These were defined as being *“…about things such as how to manage a difficult situation you or someone else is experiencing, a new activity or hobby that may improve wellbeing, cutting down on bad habits or accessing professional help. If you discussed the same issue with the same person on two different days, this counts as two different conversations”.* They were also asked to indicate their level of agreement with the statement “*I am comfortable speaking to others about their mental health or wellbeing*”, adapted from a measure previously used among farmers (7). Two additional questions assessing comfort and engagement with wellbeing-promoting activities were developed for the study: *“I am comfortable doing things to maintain or improve my own mental health or wellbeing”*, and *“In the last week, I’ve been doing things to maintain or improve my own mental health or wellbeing”*. Responses were indicated on a five-point Likert scale, where 1 = Strongly disagree and 5 = Strongly agree.

Additional questions were included in the post-campaign questionnaire to assess community members’ familiarity with the Vocal Locals campaign (rated on a five-point Likert scale, where 1 = Not at all familiar and 5 = Very familiar) and impact of the Vocal Locals campaign on their own engagement in wellbeing-related activities (yes/no response to the question *“have you done anything differently over the last couple of months because of the Vocal Locals campaign?”*). If community members responded that they had done something differently because of the Vocal Locals campaign, they were asked to describe what it was in an open text response box. Community members were also given the opportunity to write any other comments about the campaign. Postcode and gender were collected at both time points so we could ensure that the sample was comparable.

**References**

1. Andrews G, Slade T. Interpreting scores on the Kessler Psychological Distress Scale (K10). Aust N Z J Public Health. 2001;25(6):494-7. <https://doi.org/10.1111/j.1467-842x.2001.tb00310.x>

2. Keyes CL, Wissing M, Potgieter JP, Temane M, Kruger A, Van Rooy S. Evaluation of the Mental Health Continuum–Short Form (MHC–SF) in setswana‐speaking South Africans. Clinical Psychology & Psychotherapy. 2008;15(3):181-92. <https://doi.org/10.1002/cpp.572>

3. Keyes CL. The Mental Health Continuum: From languishing to flourishing in life. Journal of Health and Social Behavior. 2002:207-22. <https://doi.org/10.2307/3090197>

4. Kitchener BA, Jorm AF. Mental Health First Aid training for the public: evaluation of effects on knowledge, attitudes and helping behavior. BMC Psychiatry. 2002;2(1):10. <https://doi.org/10.1186/1471-244x-2-10>

5. Jorm AF, Kitchener BA, O'Kearney R, Dear KBG. Mental Health First Aid training of the public in a rural area: A cluster randomized trial [ISRCTN53887541]. BMC Psychiatry. 2004;4(1):33. <https://doi.org/10.1186/1471-244x-4-33>

6. Massey J, Brooks M, Burrow J. Evaluating the effectiveness of Mental Health First Aid training among student affairs staff at a Canadian university. J Stud Aff Res Pract. 2014;51(3):323-36. <https://doi.org/10.1515/jsarp-2014-0032>

7. Hagen BN, Harper SL, O'Sullivan TL, Jones-Bitton A. Tailored mental health literacy training improves mental health knowledge and confidence among Canadian farmers. Int J Environ Res Public Health. 2020;17(11). <https://doi.org/10.3390/ijerph17113807>
